# Bilateral inguinal bladder hernia following unilateral transabdominal preperitoneal repair. A case report and review of the literature

**DOI:** 10.1016/j.amsu.2019.08.005

**Published:** 2019-08-21

**Authors:** Nasser AlMohaya, Marwah Nasser E Alabdrabalameer, Khalid AlAnazi, Ahmed Mohammed AlMuhsin, Hesham Eltomy

**Affiliations:** a Department of General Surgery, King Fahd Military Medical Complex, Dhahran, Saudi Arabia; b Imam Abdulrahman Bin Faisal University, Dammam, Saudi Arabia; c Department of General Surgery, Dammam Medical Complex, Dammam, Saudi Arabia

**Keywords:** Sliding hernia, Bladder, Recurrence, Bilateral vesical hernia, Case report

## Abstract

Sliding hernia occurs when the hernia sac is partially formed by the wall of a viscus. The most common components of a sliding hernia includes the sigmoid colon, cecum, appendix, urinary bladder, and the ascending colon. However, the presence of bilateral vesical sliding hernia is rare and few cases have been reported in the literature. Patients with vesical sliding hernia may present with groin swelling with an associated lower urinary tract symptom. Computed tomography (CT) scan is the modality of choice for suspected cases which may reveal the classic pelvic micky mouse sign. The method of repair should be individualized taking in account the diagnostic findings. Although laparoscopic repair is becoming the mainstay management for inguinal hernia, the majority of sliding hernias are repaired using an open approach, which could be attributed to the presence of large hernias, associated complications, or recurrence with associated adhesions. We present a case of a 60-year-old male patient presented with bilateral inguinal swelling associated with urinary hesitancy and intermittency. He had undergone transabdominal preperitoneal (TAPP) repair for a left inguinal hernia 8 years ago. CT scan confirmed the presence of a bilateral hernia with the bladder herniating bilaterally. He underwent an elective bilateral open Lichtenstein tension-free mesh repair.

## Highlights

• Bilateral vesical hernia has been rarely reported in the literature.
• CT scan is the modality of choice for the diagnosis, which may reveal Micky mouse sign on an axial view.
• Laparoscopic and open repair have been reported for such cases.
• An open approach should be considered in large, complicated hernias, and those with extensive adhesions.

## 1 Introduction

A sliding hernia is formed when an organ protrudes (herniates) outside the abdominal cavity in such a manner that the wall of an organ and the overlying peritoneal surface constitute a side of the hernia sac [1]. Sliding hernia usually classified into three type. Type I is a partial involvement of a viscus as part of the hernia sac. Type II contains the mesentery of retroperitoneal organ and type III is referred to involvement of entire viscus into the hernia sac [1]. The frequency of sliding inguinal hernias in adults is approximately 3–5% [1]. The most common components of sliding hernia are the sigmoid colon followed by the cecum, appendix, urinary bladder and the ascending colon [1]. The bladder being a content of an inguinal hernia is rare. In one prospective study during 18 months period, inguinal sliding hernia reached only 3.4% (16/464). All patients were male. Only 2 patients (12,5%) were diagnosed with unilateral vesical sliding inguinal hernia [2]. Bilateral vesical sliding hernia is extremely rare condition. Up to our knowledge only 3 cases were reported in the literature with this rare entity [[3], [4], [5]]. We present a new case of bilateral vesical sliding hernia and managed by an open Lichtenstein tension-free mesh repair, who had previously a left transabdominal preperitoneal inguinal hernia repair (TAPP). The work has been reported in line with the SCARE criteria [6].

### 1.1 Case report

**Fig. 1:**
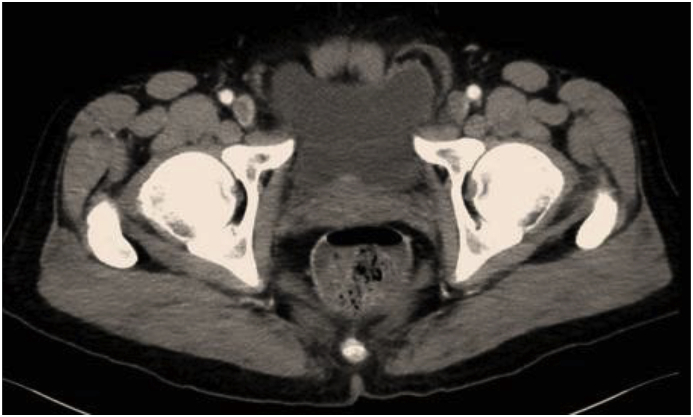
CT scan of the abdomen and pelvis showing bilateral vesical inguinal hernia, and the classic “micky mouse” sign (Axial View).

**Fig. 2:**
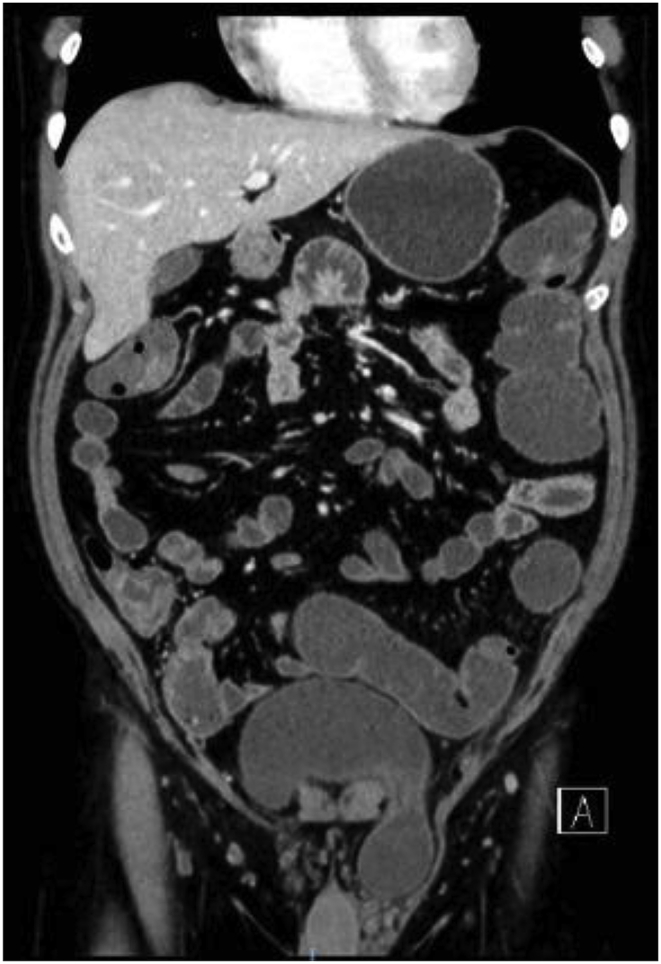
CT scan of the abdomen and pelvis showing bilateral vesical inguinal hernia, more prominent in the left side (Coronal View).

A healthy 60-year-old male not known to have any chronic medical illnesses. presented to general surgery clinic complaining of bilateral groin painless swelling, for the past 2 years. In addition, he had a history of urinary hesitancy and intermittency developed in the past few months prior to presentation. He had no other gastrointestinal or lower urinary tract symptoms. He denied any history of heavy weight lifting. He has no history of smoking. He had undergone a left transabdominal preperitoneal (TAPP) inguinal hernia repair 8 years back in another hospital. The patient is married, and works as lawyer. He is not taking any medication, and he has an unremarkable family history. Upon physical examination, the patient was well built and well nourished. He weights 78 kg, and he is 180 cm tall (BMI 24.1 kg/m2). Local examination revealed a bilateral inguinal swelling that bulged upon Valsalva maneuver. The swelling was more prominent on the left side with no evidence of scrotal extension. The urinary bladder wasn't palpable. His blood test results showed a normal complete blood count, and kidney function. Abdominal Computed tomography (CT) with contrast was obtained which showed a bilateral inguinal hernia, both containing part of the urinary bladder more evident on the left side (Pelvic micky mouse sign) (Fig. 1, Fig. 2), no bowel loops or omentum was detected in the hernia sac. Based on the clinical and radiological assessment the patient was diagnosed with bilateral bladder hernia and was planned for an elective open repair. Taking in account that the patient had a history of left TAPP inguinal hernia repair with the associated adhesion especially in the left side, and the preoperative diagnosis of bilateral sliding vesical hernia. The procedure was performed by a senior surgical resident under direct supervision of a general surgery consultant. The procedure was performed under spinal anesthesia. Intra-operatively Foley's catheter was inserted initially for urinary bladder decompression. Bilateral direct sliding inguinal hernia with solely bladder content in both hernias were identified (Fig. 1, Fig. 2). The bladder was released and reduced easily in the both sides. The floor was repaired with prolene sutures. Onlay prolene mesh patch was tailored and applied bilaterally. The procedure duration was 100 min. Blood loss was minimal. The patient had an uneventful postoperative course and was discharged on postoperative day 2. He was followed in the outpatient department for one month. No complication or evidence of recurrence was noted.

**Fig. 3:**
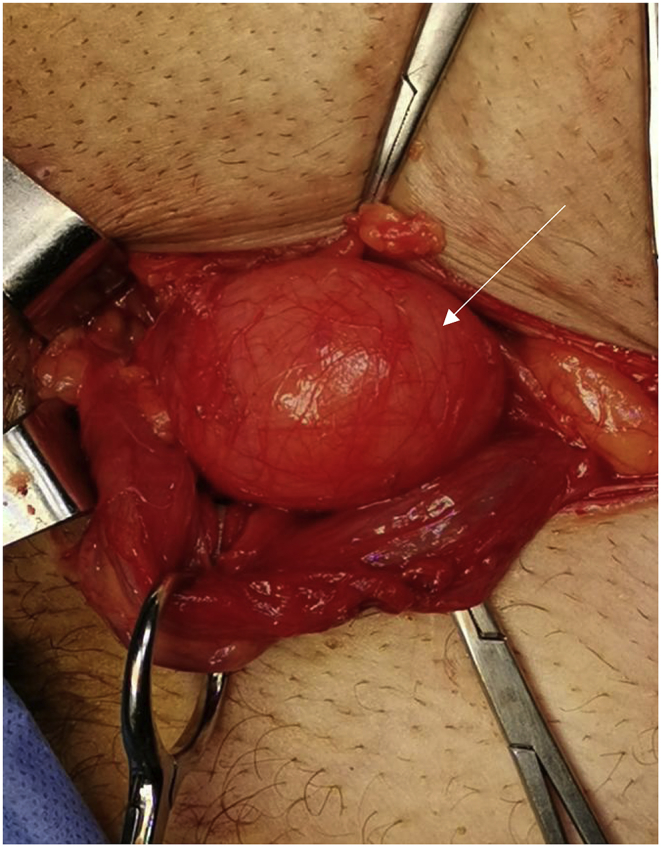
Intraoperative picture showing direct sliding hernia with bladder content in the left side (white arrow).

**Fig. 4:**
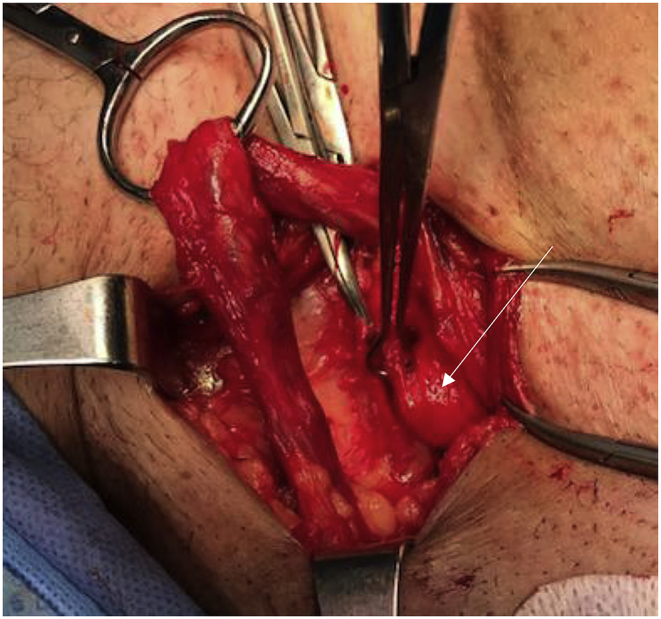
Intraoperative picture showing a partially reduced direct sliding hernia with bladder content in the right side (white arrow).

## 2 Discussion

Sliding bladder hernia is a rare type of hernia in which the wall of the bladder constitutes a part of the hernia sac. Although sliding hernia are often complicated on presentation, sliding bladder hernia are usually asymptomatic. In review of the literature, it was found that Less than 7% of bladder hernias are diagnosed preoperatively, 16% are diagnosed postoperatively owing to the associated postoperative complications, and the reminding cases are diagnosed perioperatively [1,7].

A systemic review performed in 2017 showed that most of the patients with vesical sliding inguinal hernia were males, and 76% of them were symptomatic. The symptoms were 60% inguinal swelling, 40% pain, 12.7% reduction of an inguinal mass after voiding, and 48% lower urinary tract symptoms (LUTS) which could be a voiding or storage symptoms. However, it is difficult to explain the significance of LUTS in these cases and whether it is attributed to an entrapment of herniated bladder or an underling disease like bladder outlet obstruction [8]. Kraft et al. reported 4 cases with an inguinal hernia containing bladder associated with significant LUTS. It was noted that the patient's symptoms improved markedly after hernial repair in all the reported cases [9]. Complications associated with sliding bladder hernia were rarely described in the literature, including acute renal failure or urinary retention [8]. In the presented case, the patient presented with painless bilateral groins swelling and voiding urinary symptoms in the form of hesitancy and intermittency. The diagnosis was made before the surgery with the aid of CT scan.

Preoperative diagnosis of sliding bladder hernia is essential. It allows for better surgical planning and may decrease the risk of iatrogenic injury [1]. Gomella et al. reported a 38% rate of unrecognized bladder injury in a repair of large inguinoscrotal hernias. The postoperative consequences of these injuries include sepsis, massive hematuria, and fistula formation [10].

Many different imaging modalities have been used for the diagnosis of sliding bladder hernia. Moreover, CT scan with or without contrast is the most frequent modality used [8]. It is highly sensitive in identification of the hernia and detecting the content of the hernia sac. CT scan provides the advantage of identification of other pathology or associated complications like hydronephrosis [8]. In cases of bilateral inguinal vesical hernia, it demonstrates a “Pelvic micky mouse sign” [3,4]. ultrasonography (US), and contrast-enhance retrograde cystography have also been used [8].

Various approaches have been advocated in the management of unilateral inguinal vesical hernia including open, laparoscopic, and robotic surgery. In the current literature, open surgery was the most common surgical approach adopted, performed in 80.4% of cases [8]. In cases of bilateral inguinal vesical hernia, up to our knowledge there were only 3 published case reports (Table 1). All the reported cases were males, with an average age of 53.3 years. Only one case presented with an associated LUTS symptoms. All cases were diagnosed with CT scan. The surgical management was described in one case only, which was a case of recurrent bilateral inguinal hernia managed laparoscopically with bladder takedown approach and median TAPP. The procedure duration was 132 min. The patient was discharge on postoperative day 3 [5]. Though International groin hernia guidelines recommends laparo-endoscopic repair for primary bilateral inguinal hernias, it recommends an anterior approach after recurrence of inguinal hernia post TAPP repair. Moreover, mesh-based repair is recommended for patients with inguinal hernia [11]. In the presented case, the decision was made to go for bilateral open Lichtenstein tension-free mesh repair. Taking into account that the patient had a history of left TAPP inguinal hernia repair with the associated adhesion especially in the left side, and taking into consideration the preoperative diagnosis of bilateral sliding vesical hernia. The procedure duration was 100 min. The patient was discharge home on postoperative day 2.

**Table 1:** summary of the reported cases of bilateral inguinal vesical hernia.

Author and Year	Sabharwal S. et al. (2013)	Indiran V. et al. (2016)	Umemura A. et al. (2018)	Presented case (2019)
Age/gender	50 years, male	41 years, male	69 years, male	60 years, male
Presenting symptoms	Right flank pain	Bilateral loin pain	Bilateral groin painful swelling	Bilateral groin painless swelling
LUTS	none	none	Frequent voiding	urinary hesitancy and intermittency
Past surgical history	none	none	Bilateral TAPP repair 3 years back	Left TAPP repair 8 years back
Physical examination	unremarkable	N/A	Bilateral inguinal swelling	Bilateral inguinal swelling
Diagnosis modality	CT scan	CT scan	CT scan	CT scan
Management	N/A	N/A	Laparoscopically with bladder takedown approach and median TAPP	Bilateral open Lichtenstein tension-free mesh repair
Operative time	N/A	N/A	132 min	100 min
Intraoperative blood loss	N/A	N/A	2 ml	10 ml
Postoperative Complications	N/A	N/A	None	None
Recurrence	N/A	N/A	No	No
Length of Hospital stay	N/A	N/A	Discharged on day 3 postoperative	Discharged on day 2 postoperative

NA: not available LUTS: lower urinary tract symptoms.

## 3 Conclusion

Vesical sliding inguinal hernia is a rare entity. Moreover, bilateral sliding vesical hernia has been scarcely reported in the literature. Patients with this pathology may present with an inguinal swelling associated with lower urinary tract symptoms. US and CT scan is the modality of choice for preoperative diagnosis in patient with an inguinal hernia, when a bladder involvement is suspected. Bilateral open Lichtenstein tension-free mesh repair is safe and effective option for such cases. However, the method of repair should be tailored for each individual case, taking in account careful analysis of clinical presentation, diagnostic findings, and the patient's surgical history.

## Disclosure

The authors declare that there is no conflict of interest regarding the publication of this paper.

## Provenance and peer review

Not commissioned, externally peer reviewed.

## Ethical approval

This case report is exempt from ethical approval by our institution.

## Sources of funding

This case report had no funding or sponsors.

## Consent

Written informed consent was obtained from the patient for publication of this case report and accompanying images. A copy of the written consent is available for review by the Editor-in-Chief of this journal on request.

## Author contribution

NM wrote the manuscript, reviewed the literature, and assisted in the surgery. MN drafted the manuscript. KA participated in data acquisition and reviewed the literature. AM contributed to the conception and the design of the study, reviewed the manuscript, and performed the surgery. HE supervised the management of the patient and reviewed the manuscript. All authors read and approved the final manuscript.

## Conflicts of interest

The authors declare that there is no conflict of interest regarding the publication of this paper.

## Trial registry number

None.

## Guarantor

Dr. Ahmed Mohammed Al. Muhsin

## Appendix A Supplementary data

1; scare_2018_guidelines.docx 1; scare_2018_guidelines.docx
